# Broad anti-SARS-CoV-2 antibody immunity induced by heterologous ChAdOx1/mRNA-1273 vaccination

**DOI:** 10.1126/science.abn2688

**Published:** 2022-02-10

**Authors:** Chengzi I. Kaku, Elizabeth R. Champney, Johan Normark, Marina Garcia, Carl E. Johnson, Clas Ahlm, Wanda Christ, Mrunal Sakharkar, Margaret E. Ackerman, Jonas Klingström, Mattias N. E. Forsell, Laura M. Walker

**Affiliations:** ^1^ Adimab LLC, Lebanon, NH 03766, USA.; ^2^ Thayer School of Engineering, Dartmouth College, Hanover, NH 03755, USA.; ^3^ Division of Immunology, Department of Clinical Microbiology, Umeå University, Umeå, Sweden.; ^4^ Centre for Infectious Medicine, Department of Medicine Huddinge, Karolinska Institutet, Stockholm, Sweden.; ^5^ Geisel School of Medicine, Dartmouth College, Hanover, NH 03755, USA.; ^6^ Adagio Therapeutics, Inc., Waltham, MA 02451, USA.

## Abstract

Heterologous prime-boost immunization strategies have the potential to augment COVID-19 vaccine efficacy. We longitudinally profiled SARS-CoV-2 spike (S)-specific serological and memory B cell (MBC) responses in individuals receiving either homologous (ChAdOx1:ChAdOx1) or heterologous (ChAdOx1:mRNA-1273) prime-boost vaccination. Heterologous mRNA booster immunization induced higher serum neutralizing antibody and MBC responses against SARS-CoV-2 variants of concern (VOCs) compared to homologous ChAdOx1 boosting. Specificity mapping of circulating B cells revealed that mRNA-1273 boost immunofocused ChAdOx1-primed responses onto epitopes expressed on prefusion-stabilized S. Monoclonal antibodies isolated from mRNA-1273 boosted participants displayed overall higher binding affinities and increased breadth of reactivity against VOCs relative to those isolated from ChAdOx1-boosted individuals. Overall, the results provide molecular insight into the enhanced quality of the B cell response induced following heterologous mRNA booster vaccination.

Multiple safe and effective COVID-19 vaccines have been developed at an unprecedented scale and speed. However, waning vaccine-induced immunity and the emergence of SARS-CoV-2 VOCs with increased neutralization resistance have limited the effectiveness of currently available vaccines (*1*–*3*). An early challenge in vaccine distribution was a halt in ChAdOx1 nCoV-19/AZD1222 (hereafter referred to as ChAdOx1) booster immunization in certain age groups driven by evidence of rare but serious thrombotic events (*4*, *5*). To fully immunize individuals who received a single dose of ChAdOx1 but were not eligible for a ChAdOx1 boost, several countries recommended a switch to heterologous booster vaccination with mRNA-1273 (Moderna) or BNT162b2 (Pfizer/BioNTech). This provided an opportunity to learn that heterologous ChAdOx1/mRNA prime-boost immunization induces higher serum neutralizing antibody titers and confers increased levels of protection relative to homologous ChAdOx1 dosing, but the molecular basis for this difference in immunogenicity remains unknown (*6*–*9*).

All currently available COVID-19 vaccines are based on the SARS-CoV-2 spike protein, which plays a key role in viral entry and is the primary target for neutralizing antibodies. The S protein exists on the surface of the virion in a metastable prefusion conformation, and binding of the receptor binding domain (RBD) to angiotensin converting enzyme 2 (ACE2) triggers shedding of the S1 subunit and transition of the S2 subunit to a highly stable postfusion conformation (*10*). Early structural and biochemical studies demonstrated that stabilization of the spike ectodomain via two consecutive proline substitutions in the S2 subunit (S-2P) prevents the transition from the prefusion to postfusion state and leads to enhanced immunogenicity in animal models (*11*, *12*). Several COVID-19 vaccines encode S-2P, such that the protein maintains the prefusion conformation and avoids S1 shedding (e.g., mRNA-1273, BNT162b2, and Ad26.COV2.S), whereas others express wild-type (WT) S (e.g., ChAdOx1, Sputnik V, and CoronaVac), which likely leads to expression of both pre- and post-fusion conformations of S. We comprehensively interrogated serological and circulating B cell responses induced by prime immunization with an adenovirus vector-based vaccine encoding WT S (ChAdOx1) and following a second dose of either ChAdOx1 or an mRNA vaccine expressing S-2P (mRNA-1273).

We recruited 55 healthcare workers receiving either homologous ChAdOx1:ChAdOx1 or heterologous ChAdOx1:mRNA-1273 prime-boost vaccination for blood donation (table S1). None of the volunteers had a documented history of prior SARS-CoV-2 infection. Participants received one dose of ChAdOx1 and 9-12 weeks later, a second dose of either ChAdOx1 (*n* = 28) or mRNA-1273 (*n* = 27). We collected the first blood sample on the day of booster immunization to analyze ChAdOx1-primed immune responses and a second sample 7-10 days following the second dose to study the early secondary B cell response induced by homologous or heterologous booster vaccination (Fig. 1A).

**Fig. 1.**
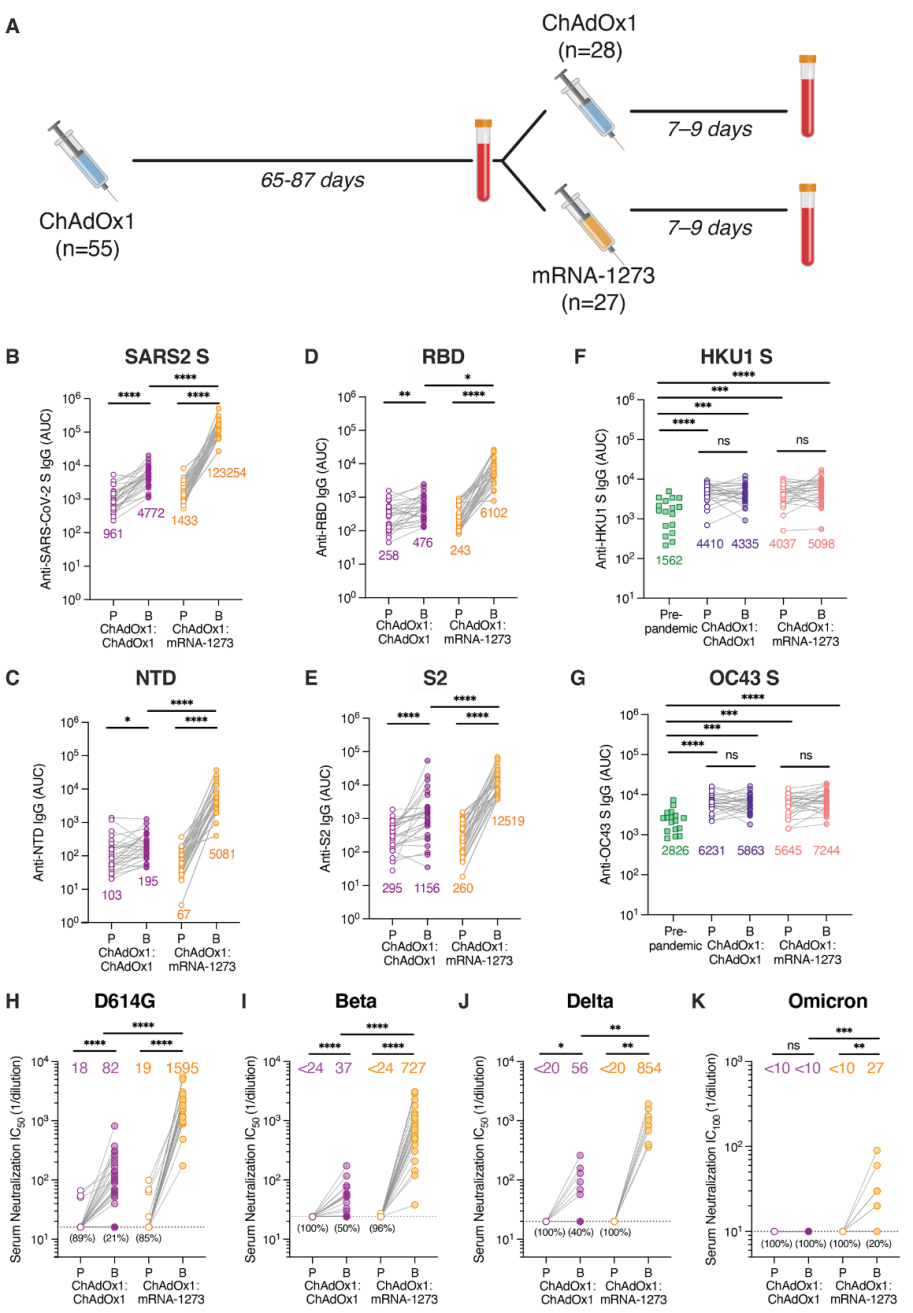
Serum binding and neutralizing activity following homologous and heterologous prime-boost vaccination. (**A**) Immunization and blood draw schedule. (**B** to **G**) Serum IgG binding to recombinant SARS-CoV-2 S (1:1 mixture of WT S and S-2P antigens) (B), NTD (C), RBD (D), prefusion-stabilized S2 (E), HKU1 S (F), and OC43 S (G), as assessed by ELISA. Binding of pre-pandemic donor sera (*n* = 17) is shown for comparison in F-G. Geometric mean AUC values are shown below data points. (**H** to **J**) Serum neutralizing activity against authentic SARS-CoV-2 D614G (H) and Beta/B.1.351 (I) measured by plaque reduction assay (*n* = 27-28 donors from each cohort), Delta/B.1.617.2 (J) measured by cytopathic effect (CPE)-based colorimetric microneutralization assay (*n* = 10 donors from each cohort). Plotted values represent 50% serum neutralizing titers. Values below the dotted line indicate the percentage of samples with serum neutralizing titers below the limit of detection. (**K**) Serum neutralizing activity against authentic Omicron/B.1.1.529/BA.1 measured by 100% CPE inhibition (*n* = 10 donors from each cohort). Values below the dotted line indicate the percentage of samples with serum neutralizing titers below the limit of detection. Statistical comparisons between prime and boost were determined by Wilcoxon pair-matched rank sum test. Statistical comparisons across groups were determined by two-tailed Mann Whitney *U* test with Bonferroni correction [(B) to (E)] and (H) to (K)] or two-sided Kruskal Wallis test by ranks with subsequent Dunn's multiple comparisons [(F) and (G)]. *P < 0.05, **P < 0.01, ***P < 0.001, ****P<0.0001. P, Prime; B, Boost; AUC; area under the curve; LOD, limit of detection; ns, non-significant. All data are representative of at least two independent experiments.

We first evaluated serum IgG binding activity at both sampling timepoints. All participants mounted weak but detectable SARS-CoV-2 S-specific serum IgG binding responses following the first dose of ChAdOx1, and homologous booster vaccination resulted in a small but significant (4.6-fold) increase in serum IgG binding antibodies (Fig. 1B and fig. S1). In contrast, heterologous booster vaccination with mRNA-1273 led to a much larger (86-fold) increase in S-specific serum IgG binding responses (Fig. 1B and fig. S1). Correspondingly, ChAdOx1 prime immunization elicited weak serum IgG binding activity to recombinant receptor binding domain (RBD), N-terminal domain (NTD), and prefusion-stabilized S2 subdomains (geometric mean AUCs ranging 67-321), and homologous and heterologous booster immunization enhanced these responses by 2-4-fold and 25-77-fold, respectively (Fig. 1, C to E).

Cross-reactive MBCs induced by seasonal β-CoVs are activated and expanded following primary SARS-CoV-2 infection and mRNA vaccination (*13*, *14*). To determine whether ChAdOx1:ChAdOx1 and/or ChAdOx1:mRNA-1273 vaccination elicited similar recall responses, we assessed serum IgG binding to recombinant OC43 and HKU1 S proteins at both sampling time points. At the pre-boost time point, we detected significantly elevated serum antibody titers to both OC43 and HKU1 relative to pre-pandemic sera samples, suggesting a “back-boosting” of pre-existing cross-reactive MBCs induced by seasonal β-CoV infections (Fig. 1, F and G). However, this response was not further amplified following homologous or heterologous booster immunization.

We next evaluated serum neutralizing activity against authentic SARS-CoV-2 D614G, B.1.617.2/Delta, B.1.351/Beta, and B.1.1.529/Omicron variants at both sampling timepoints. At the pre-boost timepoint, the vast majority of donors (87%) displayed weak or undetectable serum neutralizing activity against SARS-CoV-2 D614G, with 50% neutralization titers ranging from <16 to 66 (Fig. 1H), and none of the donors showed detectable serum neutralization against Beta, Delta, or Omicron (Fig. 1, I to K). Homologous ChAdOx1 booster immunization resulted in an overall 4.6-fold increase in serum neutralizing activity against SARS-CoV-2 D614G (geometric mean titer = 82), but neutralizing titers remained at the lower limit of detection in 21% (6/28) of donors (Fig. 1H). In about half of the individuals, we also observed a small but significant (1.5 to 2.4-fold) rise in serum neutralizing activity against Beta and Delta following ChAdOx1 booster immunization (Fig. 1, I and J), but none of the donor sera exhibited detectable neutralization against Omicron (Fig. 1K). In contrast to the relatively weak serum neutralizing responses observed following two doses of ChAdOx1, heterologous mRNA-1273 booster vaccination significantly enhanced serum neutralizing titers to SARS-CoV-2 D614G, Beta, and Delta in all donors (geometric mean titers = 1595, 727, 854 for D614G, Beta, Delta, respectively) (Fig. 1, H to J). Furthermore, about 80% of donor sera displayed low but detectable neutralizing activity against Omicron, with 100% neutralization titers ranging from <10 to 90 (Fig. 1K). Taken together, heterologous ChAdOx1:mRNA-1273 prime-boost vaccination elicits superior binding and neutralizing antibody responses to SARS-CoV-2 and VOCs relative to homologous ChAdOx1:ChAdOx1 vaccination.

Previous studies of other viral infections have shown that booster vaccination typically induces a rapid and robust antigen-specific plasmablast response that peaks at approximately day 7 following immunization and accounts for a relatively large proportion of peripheral blood B cells (*15*, *16*). We therefore assessed the frequency of plasmablasts in peripheral blood 7-10 days following homologous and heterologous booster vaccination (fig. S2A). Although we observed modest plasmablast expansion in a subset of ChAdOx1 boosted donors, the median frequency of plasmablasts across all donors was not significantly elevated relative to pre-boost or unpaired pre-pandemic healthy donor samples (Fig. 2A). In contrast, heterologous booster immunization induced a robust plasmablast response in almost all donors, where plasmablast frequencies averaged 3% and accounted for up to 7% of all CD19^+^ peripheral blood B cells (Fig. 2A).

**Fig. 2.**
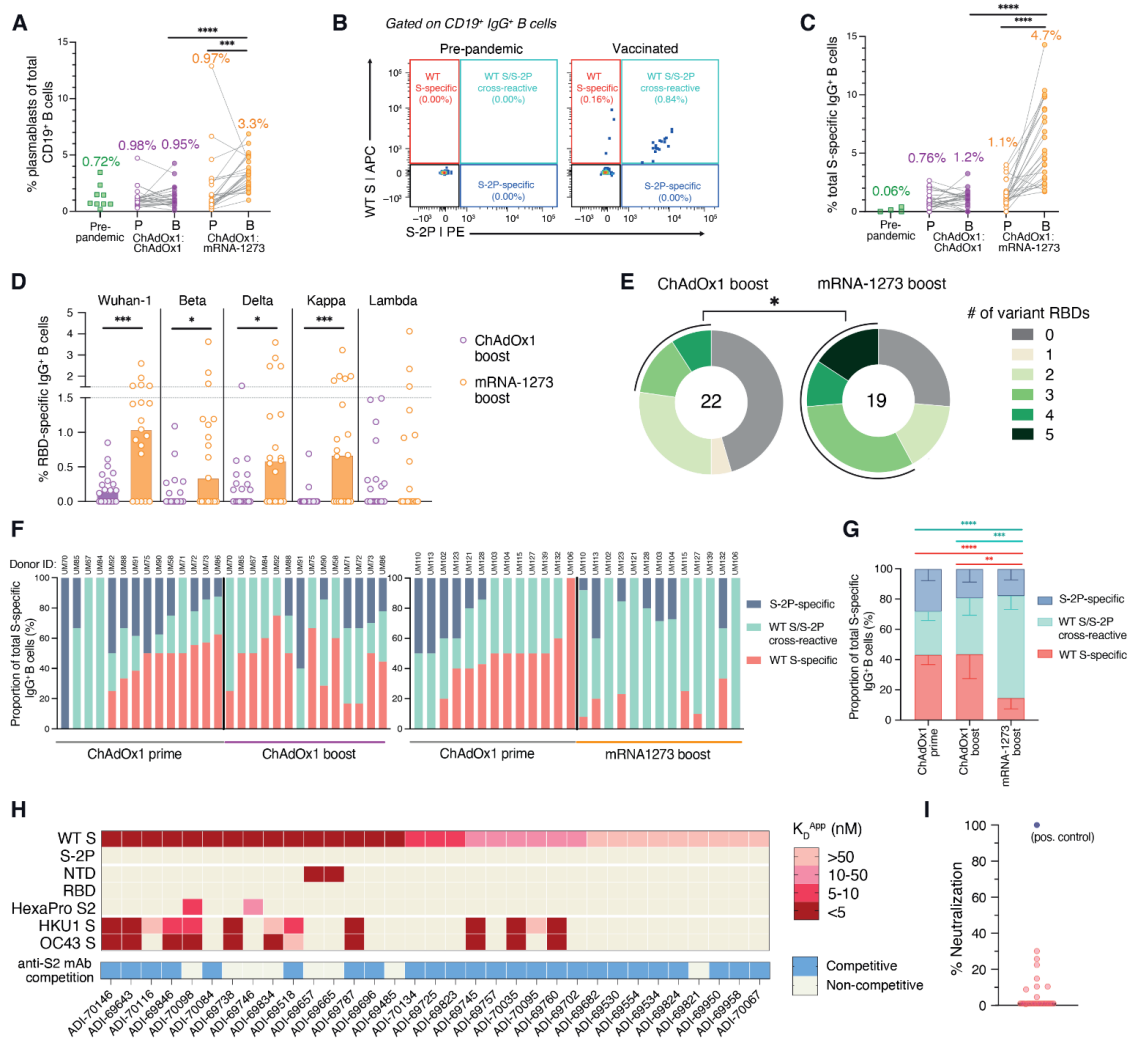
SARS-CoV-2 S-specific B cell responses induced by homologous and heterologous prime-boost vaccination. (**A**) Frequencies of plasmablasts (defined as CD19^+^CD20^—/lo^CD71^+^ cells) among circulating CD19^+^ B cells following prime and boost vaccination, as determined by flow cytometry. Pre-pandemic donor samples (*n* = 9) were included for comparison. Median values are shown above data points. (**B**) Representative fluorescence-activated cell sorting (FACS) gating strategy used for identifying WT S-specific, S-2P-specific, and WT/S-2P cross-reactive IgG^+^ B cells. (**C**) Frequencies of total (WT + S-2P) SARS-CoV-2 S-reactive B cells among circulating IgG^+^ B cells, as determined by flow cytometry. Median frequencies are shown above data points. (**D**) Frequency of circulating IgG^+^ B cells reactive with RBDs encoding mutations present in Beta, Delta, Kappa, and Lambda variants. The height of each bar indicates median frequency. (**E**) Proportion of donors with detectable B cell reactivity with the indicated number of variant RBDs. The total number of donors analyzed is indicated in the center of the pies. Statistical significance was determined by Fisher's exact test and calculated based on the proportion of donors with B cells displaying reactivity to ≥3 variant RBDs. (**F**) Proportions of WT S-specific, WT S/S-2P cross-reactive, and S-2P-specific B cells among total S-specific B cells following homologous (left) or heterologous (right) prime-boost immunization. Donors with S-specific B cell frequencies <1% of total IgG^+^ B cells at either time point were excluded from this analysis. Donor IDs are denoted above each bar. (**G**) Mean proportions of WT S-specific, WT S/S-2P cross-reactive, and S-2P-specific B cells across all donors within each cohort. Error bars indicate 95% confidence intervals. (**H**) Apparent binding affinities (K_D_^App^) of WT S-specific monoclonal antibodies for WT S, S-2P, prefusion S subdomains (NTD, RBD, prefusion-stabilized S2), HKU1 S, and OC43 S, as determined by biolayer interferometry (BLI). Competitive binding with an anti-S2 antibody (ADI-69962), as determined by a BLI competitive sandwich assay, is indicated below the heatmap. (**I**) Neutralizing activity of WT S-specific antibodies against MLV-SARS-CoV-2 Wuhan-1 at a concentration of 1 μg/ml. A previously described anti-RBD neutralizing antibody (ADG-2) was included as a positive control (*29*). Statistical comparisons between paired prime and boost samples were determined by [(A) and (C)] Wilcoxon pair-matched rank sum test. Statistical comparisons between vaccination cohorts were determined by two-sided Mann-Whitney *U* tests (D) and two-sided Kruskal-Wallis test by ranks with subsequent Dunn's multiple comparisons [(C) and (G)]. *P < 0.05, **P < 0.01, ***P < 0.001, ****P < 0.0001. P, Prime; B, Boost; *K*_D_^App^; apparent equilibrium constant.

We next investigated the magnitude and specificities of the S-specific MBC response induced following both prime and boost immunization. Because ChAdOx1 encodes WT S, which may elicit B cell responses to both pre- and post-fusion conformations of S, we evaluated B cell responses to both prefusion-stabilized (S-2P) and WT (unstabilized) forms of the S protein (Fig. 2B and fig. S2B). SDS-PAGE analysis revealed that the recombinant WT S preparation used for B cell staining contained both uncleaved and cleaved forms of S, the latter likely representing a mixture of prefusion, postfusion, and non-native or dissociated forms of S (fig. S3). The frequency of total S (WT + S-2P)-specific IgG^+^ B cells ranged from 0-3.7% following ChAdOx1 prime immunization, and homologous booster immunization did not significantly enhance this response in most donors (Fig. 2C). In contrast, mRNA-1273 booster vaccination induced a robust expansion of S-specific IgG^+^ B cells in most donors, averaging a 4-fold increase over the corresponding ChAdOx1 prime-induced responses (Fig. 2C). Similarly, mRNA-1273 booster immunization expanded S-specific IgM^+^CD27^+^ and IgA^+^ MBC cell populations (fig. S4). mRNA-1273 boost also elicited significantly higher magnitude B cell responses to individual subdomains within prefusion S (RBD, NTD, and prefusion-stabilized S2), including RBDs encoding mutations found in the Beta, Delta, and Kappa variants, relative to homologous ChAdOx1 boost (Fig. 2D and figs. S5 and S6). Only 23% of ChAdOx1 boosted donors displayed detectable B cell reactivity with ≥3 variant RBDs compared to 58% of mRNA boosted donors (Fig. 2E). Overall, the results suggest that heterologous ChAdOx1:mRNA-1273 prime-boost vaccination induces a more robust B cell response to SARS-CoV-2 and VOCs relative to homologous ChAdOx1:ChAdOx1 immunization.

The use of dual-labeled SARS-CoV-2 S probes for B cell staining allowed us to assess the frequencies and proportions of WT S-specific, WT S/S-2P-reactive, and S-2P-specific B cells induced before and after booster immunization. In the majority of donors (19/27), ≥30% of the total S-specific IgG^+^ MBC response induced by ChAdOx1 prime immunization was directed toward epitopes expressed only on WT S, with the remaining B cells displaying either specificity for S-2P or cross-reactivity between WT S and S-2P (Fig. 2, F and G). As expected, homologous ChAdOx1 booster vaccination did not significantly modify this response in most donors (Fig. 2, F and G). In contrast, we observed a massive decline in the proportion of circulating WT S-specific B cells following mRNA booster immunization in most donors (Fig. 2, F and G). Correspondingly, booster immunization with mRNA-1273 preferentially expanded WT S/S-2P cross-reactive B cells, increasing from a median frequency of 0.3% prior to booster immunization to 3.4% following mRNA-1273 boost (fig. S7). Thus, in addition to driving a robust expansion of ChAdOx1 prime-induced B cells, heterologous mRNA-1273 booster immunization re-directs the B cell response toward epitopes expressed on prefusion S.

To characterize the specificities and functional properties of the WT S-specific antibodies induced by ChAdOx1 prime immunization, we cloned and expressed 33 monoclonal antibodies from single WT S-specific B cells in five donors. The antibodies utilized a diversity of variable heavy- (VH) and light-chain (VL) germline genes, and 28 out of 33 contained somatic mutations, consistent with an MBC origin (fig. S8). The avid binding affinities of the antibodies for recombinant WT S ranged from 1 to 36 nM and none displayed detectable binding to S-2P (Fig. 2H). Over 85% (29/33) of the WT S-specific antibodies failed to bind to recombinant subdomains comprising prefusion S and 40% displayed reactivity with HKU1 and/or OC43 S, potentially suggesting recognition of conserved epitopes within postfusion S2 (Fig. 2H). Given the lack of availability of recombinant postfusion S antigens, we evaluated this possibility by performing competitive binding assays with an anti-S2 antibody (ADI-69962) that targets an epitope expressed on WT S, S-2P and prefusion-stabilized S2 antigens (fig. S9). 76% (25/33) of the WT S-specific antibodies showed competitive binding with ADI-69962, suggesting recognition of a distinct antigenic site within the S2 subunit that overlaps with the ADI-69962 epitope but is not expressed on S-2P (Fig. 2H). Consistent with their lack of reactivity with prefusion S, none of the WT S-specific antibodies displayed >50% neutralizing activity against SARS-CoV-2 Wuhan-1 at a 1 μg/ml concentration (Fig. 2I). We conclude that a relatively large proportion of the SARS-CoV-2 S-specific B cell response induced by homologous ChAdOx1 prime-boost immunization is comprised of non-neutralizing anti-S2 specificities that fail to bind prefusion S.

To determine whether homologous and heterologous booster vaccination regimens induce distinct B cell responses to prefusion S, we obtained 163 and 252 paired VH and VL sequences from single S-2P-reactive B cells from 4 donors in each cohort following ChAdOx1 or mRNA-1273 booster immunization (table S2). Both booster regimens induced highly diverse B cell responses, with 0% to 8.6% of antibodies belonging to expanded clonal lineages (fig. S10). IGHV3-30 was significantly over-represented in the S-reactive MBC population in both ChAdOx1 and mRNA-1273 boosted individuals, as observed previously in antibodies isolated from naturally infected and mRNA vaccinated donors (*17*, *18*) (fig. S11A). The antibodies isolated from both donor cohorts also displayed similar levels of somatic hypermutation (SHM), which ranged from a median of 4-7 and 6-7 VH nucleotide substitutions for ChAdOx1 and mRNA-1273 boosted donors, respectively (fig. S12A). In 7 out of 8 donors, >90% of sequences contained somatic mutations, suggesting that the acute B cell response induced by either booster regimen was largely comprised of pre-existing MBCs primed by ChAdOx1 immunization (fig. S12B). Furthermore, the degree of SHM was comparable to that observed in antibodies previously isolated from SARS-CoV-2 convalescent individuals at a similar time point following infection (approximately 3 months), suggesting that the kinetics of affinity maturation following ChAdOx1 prime immunization may be similar to that of natural infection (fig. S12). Overall, the results demonstrate that the genetic features of S-2P-reactive antibodies induced by homologous and heterologous booster vaccination are highly similar, with both groups rich in clonally expanded and somatically mutated sequences.

We next measured the binding affinities and neutralizing activities of the isolated S-2P-reactive antibodies. Antibodies isolated from mRNA boosted donors exhibited overall higher Fab binding affinities (median K_D_ = 12 nM) compared to those isolated from ChAdOx1 boosted donors (median K_D_ >100 nM) (Fig. 3A). However, only a small subset of binding antibodies isolated from both donor cohorts (6-13% and 8-21% for ChAdOx1 and mRNA-1273 boosted donors, respectively) displayed >50% neutralizing activity against SARS-CoV-2 Wuhan-1 at a concentration of 1 μg/ml (Fig. 3B). Thus, the neutralizing antibody response represents only a minor fraction of the total prefusion S-reactive binding response induced by both homologous and heterologous prime-boost immunization.

**Fig. 3.**
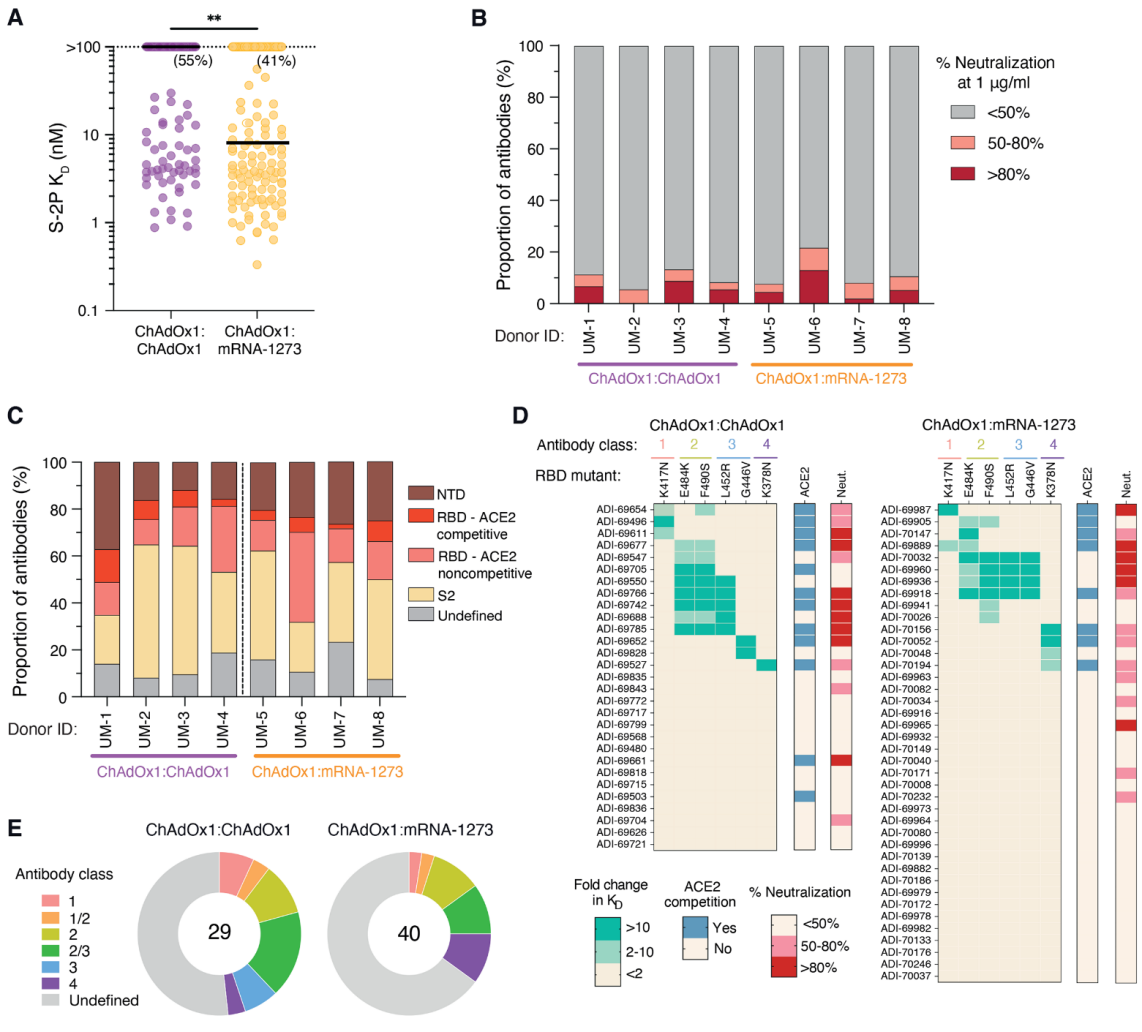
Binding and neutralization properties of monoclonal antibodies isolated from ChAdOx1 and mRNA-1273 boosted donors. (**A**) Fab binding affinities for S-2P, as determined by BLI. Antibodies with no detectable monovalent binding activity are excluded and those with weak binding affinities that could not be fit to a 1:1 binding model are plotted as *K*_D_ >100 nM. Black bars indicate medians. Values in parentheses indicate the percentage of antibodies with K_D_ >100 nM. (**B**) Proportion of antibodies exhibiting <50%, 50-80%, and >80% neutralization activity against MLV-SARS-CoV-2 Wuhan-1 at a concentration of 1 μg/ml. (**C**) Proportion of S-2P-reactive antibodies directed to each of the indicated subdomains within prefusion S. Competitive hACE2 binding was determined using a BLI-based competition assay. (**D**) Heatmaps displaying fold change in binding affinity of anti-RBD antibodies to variant RBDs containing the indicated single point mutations. Competitive hACE2 binding activity and percentage neutralization against MLV-SARS-CoV-2 Wuhan-1 at a concentration of 1 μg/ml are shown in the bars on the right. (**E**) Summary of the distribution of anti-RBD antibodies belonging to each of the indicated classes. The numbers in the center of the pies indicate the total number of antibodies analyzed. Statistical comparisons were made by (A) two-sided Mann-Whitney *U* tests. *K*_D,_ equilibrium dissociation constant. **P < 0.01.

To examine how the type of booster immunization impacts the B cell immunodominance hierarchy to prefusion S, we evaluated the proportion of S-2P-reactive antibodies directed to the NTD, RBD, and prefusion-stabilized S2 subdomains in each donor from which monoclonal antibodies were isolated. We observed relatively similar proportions of antibodies targeting each subdomain within prefusion S, although S2-directed antibodies dominated the response in a subset of donors in both groups (Fig. 3C). S-2P-reactive antibodies displaying cross-reactivity with OC43 and HKU1 S only comprised about 5% of the S-2P-reactive B cell response elicited by both booster regimens (fig. S13). RBD-directed hACE2-blocking antibodies also represented a small proportion of the S-2P-reactive binding response in both donor cohorts, ranging from 3-14% and 2-9% in ChAdOx1:ChAdOx1 and ChAdOx1:mRNA-1273 immunized donors, respectively (Fig. 3C). As expected, these rare ACE2-competitive antibodies represented the majority of the neutralizing response, thus explaining the limited number of neutralizing antibodies observed among total S-2P binding antibodies (fig. S14). To further map the epitopes recognized by the RBD-directed antibodies, we evaluated their binding reactivities with recombinant RBDs containing mutations associated with escape from common antibody classes, including K417N (class 1), E484K and F490S (class 2), L452R and G446V (class 3), and K378N (class 4) (*19*, *20*). Both booster regimens induced comparable proportions of antibodies targeting common antigenic sites within the RBD (Fig. 3, D and E). In conclusion, although homologous ChAdOx1 booster vaccination induces a higher frequency of WT S-specific antibodies compared to heterologous mRNA-1273 boost, both immunization regimens establish similar immunodominance hierarchies to prefusion-stabilized S.

Finally, we assessed the RBD- and NTD-directed antibodies isolated from both donor cohorts for reactivity with multiple SARS-CoV-2 VOCs and variants of interest (VOIs). Against pre-Omicron VOCs/VOIs (Beta, Gamma, Delta, Kappa, and Lambda), 41% (12/29) of anti-RBD antibodies derived from ChAdOx1 boosted individuals displayed reduced binding activity (>2-fold) to two or more of these variants as compared to only 20% (8/40) of antibodies isolated from mRNA-1273 boosted individuals, suggesting that mRNA-1273 booster vaccination activates a larger proportion of broadly reactive B cells (Fig. 4A and fig. S15A). Furthermore, the mRNA-elicited antibodies bound to both WT and pre-Omicron VOC/VOI RBDs with significantly higher affinities (median *K*_D_=1.4-3.7 nM) relative to the ChAdOx1-induced antibodies (median *K*_D_=4.9-20.3 nM) (Fig. 4B), potentially explaining their increased breadth of binding. However, in contrast to earlier variants, the Omicron variant broadly escaped recognition by RBD-directed antibodies induced by both booster regimens, with 24/29 (83%) and 29/40 (73%) antibodies derived from ChAdOx1 and mRNA-1273 boosted individuals, respectively, showing significantly reduced binding reactivity (>10-fold) to the Omicron RBD (Fig. 4A). Strikingly, among the 26 neutralizing antibodies identified from both donor cohorts, only three maintained binding to the Omicron RBD within 10-fold of the Wuhan-1 RBD (Fig. 4C). This result is consistent with the large number of mutations within the frequently targeted class 1/2/3 RBD epitopes and provides a molecular explanation for the extensive resistance of this variant to neutralization by convalescent and vaccinee sera (*21*, *22*).

**Fig. 4.**
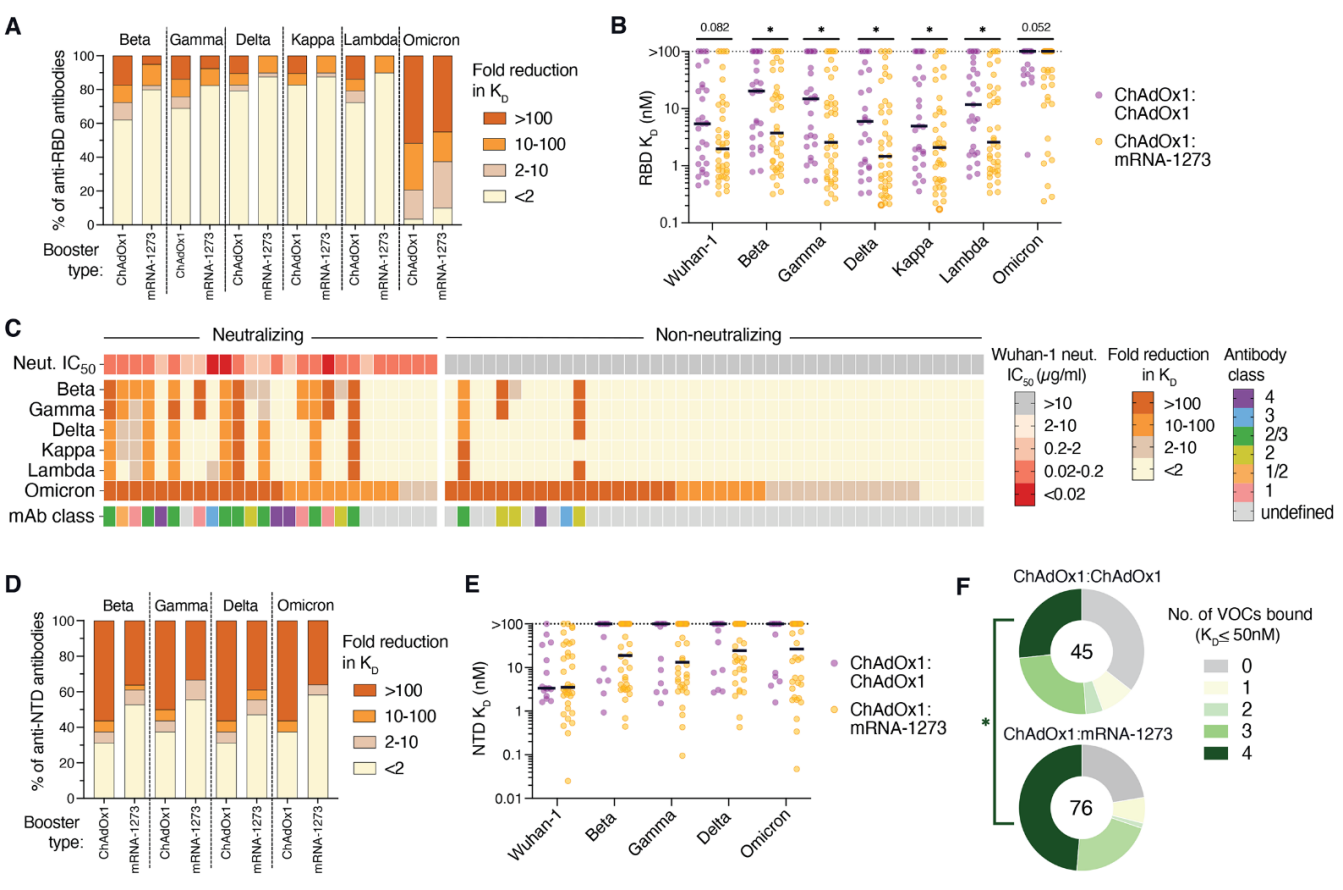
Breadth of antibody binding to SARS-CoV-2 variants. (**A**) Proportion of anti-RBD antibodies with the indicated fold reduction in Fab binding affinity to RBDs incorporating mutations present in the Beta, Gamma, Delta, Kappa, Lambda, and Omicron variants relative to the Wuhan-1 RBD, as determined by a bead-based flow cytometric assay. (**B**) Fab binding affinities of anti-RBD antibodies to Wuhan-1 and variant RBDs. Antibodies that did not reach half-maximal saturation at the highest concentration tested (100 nM) are shown as *K*_D_ >100 nM. Black bars denote medians. (**C**) Heatmap showing the fold reduction in affinity to variant RBDs compared to the Wuhan-1 RBD among neutralizing and non-neutralizing anti-RBD antibodies. The top bar indicates the neutralization IC_50_ of each mAb against MLV-SARS-CoV-2 Wuhan-1. Antibodies that did not reach 50% neutralization at 10 μg/ml were classified as non-neutralizing. The bottom bar denotes the class of each anti-RBD antibody. (**D**) Proportion of NTD-directed antibodies with the indicated fold reductions in binding activity to variant NTDs relative to the Wuhan-1 NTD. (**E**) Fab binding affinities of anti-NTD antibodies to Wuhan-1 and variant NTDs. Antibodies that did not reach half-maximal saturation at 100 nM are shown as K_D_ >100 nM. Black bars denote medians. (**F**) Combined proportions of anti-RBD and anti-NTD antibodies that bound the indicated number of variants of concern (Beta, Gamma, Delta, and Omicron) with K_D_ ≤50 nM. The number in the center of the pie indicates the total number of antibodies tested. Statistical significance was determined by [(B) and (E)] two-sided Mann-Whitney U tests or (F) Fisher's exact test. N.B., non-binding; *K*_D,_ equilibrium dissociation constant; IC_50_, 50% inhibitory concentration. *P < 0.05.

Consistent with the overall increased breadth of recognition by RBD-directed antibodies induced by heterologous booster vaccination, 52-58% of NTD-directed antibodies isolated from mRNA-1273 boosted donors retained binding to each of the four VOCs tested (Beta, Gamma, Delta, and Omicron) compared to only 31-37% of antibodies isolated from ChAdOx1 boosted donors (Fig. 4D and fig. S15B). Furthermore, the NTD-specific antibodies isolated from mRNA-1273 boosted donors trended toward higher binding affinities across all VOCs tested compared to antibodies derived from ChAdOx1 boosted donors (Fig. 4E). Overall, a significantly larger fraction of anti-RBD and -NTD antibodies (49%) derived from mRNA boosted donors retained reactivity (K_D_ < 50nM) with all VOC NTDs or RBDs relative to ChAdOx1 boosted donors (26%) (Fig. 4F). Thus, heterologous mRNA-1273 immunization appears to skew the early secondary B cell response toward higher affinity clones with improved breadth of variant recognition compared to homologous ChAdOx1 immunization, although the Omicron variant broadly escapes neutralizing antibodies induced by both booster regimens.

In conclusion, heterologous ChAdOx1:mRNA-1273 prime-boost immunization induces significantly broader and more potent serum neutralizing antibody and MBC responses against WT SARS-CoV-2 and VOCs relative to homologous ChAdOx1 vaccination, and this difference appears to be driven by both the magnitude and quality of the early secondary B cell response. Expression of WT S by ChAdOx1 appears to distract the B cell response away from neutralizing sites of vulnerability present on prefusion S, and homologous booster vaccination further expands these non-neutralizing specificities. In contrast, heterologous booster immunization with mRNA-1273, which encodes S-2P, re-directs the B cell response toward epitopes expressed on prefusion-stabilized S. Furthermore, mRNA-1273 activates B cells with higher affinity for prefusion S and greater breadth of reactivity relative to ChAdOx1. The molecular basis for this difference remains to be determined but could potentially be associated with differences in cell-surface expression of S-2P relative to WT S or the distinct innate immunostimulatory properties of mRNA versus adenoviral particles (*23*, *24*). Finally, although heterologous ChAdOx1:mRNA-1273 prime-boost immunization shows superior immunogenicity relative to two-dose ChAdOx1, the B cell response induced by both vaccination regimens is dominated by non-neutralizing antibodies and the vast majority of neutralizing antibodies fail to recognize the Omicron variant. Although studies in animal models have demonstrated that non-neutralizing antibodies can contribute to protection, serum neutralizing antibody titer strongly correlates with vaccine-induced efficacy against symptomatic COVID-19 in humans (*25*, *26*). Thus, rationally designed immunogens that focus the B cell response on conserved, neutralizing epitopes within the RBD and S2 subunit may enhance the potency, breath, and durability of protection against SARS-CoV-2, future emerging VOCs, and potentially pre-emergent betacoronaviruses (*27*, *28*).

## Supplementary Materials

### This PDF file includes:

Materials and Methods Figs. S1 to S15 Table S1 and S2 References (*30*–*35*) MDAR Reproducibility Checklist

### Other Supplementary Material for this manuscript includes the following:

MDAR Reproducibility Checklist
